# Non-Nodular CT Patterns of Solid Lung Cancer in the Early Growth Phase: A Two-Center Retrospective Study

**DOI:** 10.3390/diagnostics16203332

**Published:** 2026-10-14

**Authors:** Xiao-Feng Wu, Xiao-Dong Ge, Ting Li, Qing-Yan Zhong, Fa-Jin Lv, Zhi-Gang Chu

**Affiliations:** 1 Department of Radiology, the First Affiliated Hospital of Chongqing Medical University, No. 1 Youyi Road, Yuzhong District, Chongqing 400016, China; jeweing@126.com (X.-F.W.); 18048960342@163.com (T.L.); 13438673453@163.com (Q.-Y.Z.); 2 Department of Radiology, the Second Affiliated Hospital of Army Medical University, Chongqing 400037, China; gexdysf@126.com

**Keywords:** lung neoplasm, tomography, X-ray computed, diagnostic imaging

## Abstract

**Background/Objectives:** Early diagnosis of solid lung cancer (SLC) is essential for better prognosis. However, non-nodular patterns, atypical CT manifestations of SLC in the early growth phase, are easily overlooked, potentially leading to delayed diagnosis. This study aimed to characterize the clinical features and CT findings of non-nodular patterns of SLC in the early growth phase. **Methods:** Between January 2015 and October 2025, 365 patients with 365 pathologically confirmed SLCs were retrospectively enrolled from two centers. Based on initial morphological appearances, lesions were classified into nodular and non-nodular groups (patch-like, strip-like, bead-like, pleural plaque-like, and cystic-cavity subtypes). Clinical data and CT findings were summarized and compared between the two groups. **Results:** Of the 365 SLCs, 107 (29.32%) initially presented as non-nodular lesions, among which patch-like lesions (56, 52.34%) were the most prevalent, followed by bead-like (20, 18.69%), strip-like (16, 14.95%), pleural plaque-like (9, 8.41%), and cystic-cavity lesions (6, 5.61%). Both nodular and non-nodular lesions were predominantly located in the upper lobes (55.04% vs. 58.88%, *p* = 0.611). Multivariable analysis revealed that smoking history (adjusted odds ratio [aOR], 2.93; 95% confidence interval [CI], 1.05–8.28; *p* = 0.040), subpleural distribution (aOR, 3.37; 95% CI, 1.61–7.72; *p* < 0.001), interstitial lung disease (ILD) (aOR, 66.45; 95% CI, 23.57–220.04; *p* < 0.001), and post-tuberculosis lung changes (aOR, 8.60; 95% CI, 2.37–32.64; *p* = 0.001) were independently associated with a non-nodular pattern (AUC: 0.869; *p* < 0.001). **Conclusions:** In smokers or patients with ILD/post-tuberculosis lung changes, subpleural non-nodular lesions in the upper lobes require close monitoring for growth.

## 1. Introduction

Lung cancer is the leading cause of cancer-related death worldwide [1]. On CT images, lung cancers can be broadly categorized as pure solid or subsolid lesions according to the presence of a ground-glass opacity (GGO) component [2]. Compared with subsolid lung cancers, solid lung cancers (SLCs) are generally more aggressive and have a higher risk of recurrence and a poorer prognosis [3,4]. Therefore, early detection and diagnosis of SLC are essential for improving clinical outcomes.

In the early growth phase, SLCs typically manifest as nodules, which have long been a major focus of research. Even small nodules lacking malignant features are routinely monitored so that interval growth or newly emerging malignant characteristics can facilitate timely diagnosis [2,5,6,7]. In clinical practice, however, some SLCs do not present as nodules. Owing to the absence of typical established radiological features of lung cancer, these non-nodular lesions are not only challenging to identify accurately but also prone to being misdiagnosed as benign lesions. Consequently, they fail to attract sufficient clinical attention, leading to delayed or neglected surveillance and, ultimately, missed opportunities for optimal surgical intervention.

In recent years, studies on the diagnosis and differential diagnosis of early-stage SLCs have predominantly focused on identifying independent CT predictors, dynamic imaging changes, and artificial intelligence-based models [8,9,10,11]. Nevertheless, nearly all such investigations originate from nodules, while non-nodular lesions of SLCs remain understudied. Accordingly, the radiological spectrum of early SLCs remains incompletely defined, and current diagnostic, screening, and assessment frameworks provide limited guidance for recognizing these atypical non-nodular lesions, which therefore warrant further investigation.

In this study, we retrospectively analyzed serial preoperative chest CT data from patients with pathologically confirmed SLCs. We classified the non-nodular lesions based on morphological appearances on initial CT scans and compared clinical and radiological features between the nodular and non-nodular groups, aiming to characterize these non-nodular patterns in order to facilitate early recognition of potential SLCs.

## 2. Materials and Methods

### 2.1. Study Design, Setting, and Participants

This retrospective study was approved by the Institutional Review Boards of the First Affiliated Hospital of Chongqing Medical University (No. 2022-K90; approval date: 3 March 2022) and the Second Affiliated Hospital of Army Medical University (No. 2022-196-01; approval date: 21 June 2022), and the requirement for informed consent was waived. The study was conducted in accordance with the Declaration of Helsinki (as revised in 2013).

A total of 15,245 patients with pathologically confirmed pulmonary lesions who underwent at least two chest CT scans were initially recruited from the First Affiliated Hospital of Chongqing Medical University (Center 1; *n* = 14,316, between January 2015 and October 2025) and the Second Affiliated Hospital of Army Medical University (Center 2; *n* = 929, between May 2021 and June 2025). The diagnostic breakdown was primary peripheral lung cancer (*n* = 8164), primary central lung cancer (*n* = 2986), benign pulmonary tumors (*n* = 512), pulmonary metastases (*n* = 767), and other benign pulmonary lesions (*n* = 2816).

Among patients with primary peripheral lung cancer, exclusions were a subsolid appearance (pure ground-glass or part-solid) on the latest CT (*n* = 4665), an interval of <6 months between the initial and latest CT examinations before surgical resection or biopsy (*n* = 2788), or multiple pulmonary lesions (*n* = 106). This left 605 patients (Center 1, *n* = 529; Center 2, *n* = 76) with a pure solid nodule or mass on the latest CT.

**Figure 1:**
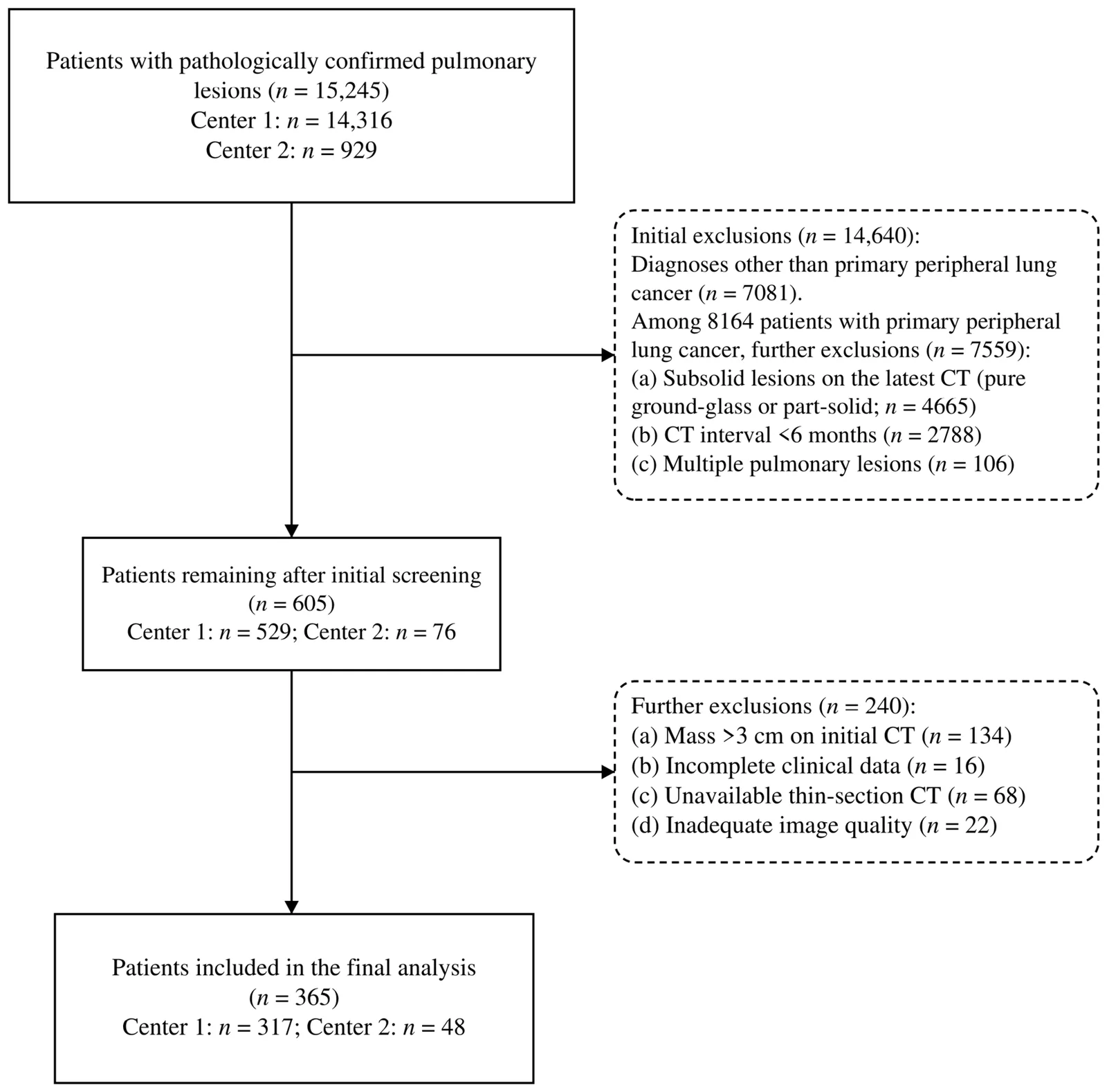
Flowchart of patient selection. CT, computed tomography.

A further 240 patients were excluded because of a lesion >3 cm on the initial CT (*n* = 134), incomplete clinical data (*n* = 16), unavailable thin-section CT images (*n* = 68), or image quality inadequate for radiological evaluation (*n* = 22). The final cohort comprised 365 patients (Center 1, *n* = 317; Center 2, *n* = 48), each contributing one lesion (Figure 1).

The minimum 6-month interval was selected as a pragmatic window for assessing morphological evolution [12] and was not a recommended clinical follow-up interval [13]. The initial 3-cm limit focused the study on smaller lesions before mass formation, consistent with the conventional nodule–mass distinction [14]. Lesion size on the latest CT was unrestricted.

### 2.2. CT Acquisition

All patients underwent chest CT examinations on one of the following scanners: SOMATOM Perspective (Siemens Healthineers, Erlangen, Germany), SOMATOM Force (Siemens Healthineers, Erlangen, Germany), Discovery CT750 HD (GE Healthcare, Milwaukee, WI, USA), Aquilion ONE PURE ViSION (Canon Medical Systems, Tokyo, Japan), SOMATOM Definition Flash (Siemens Healthineers, Erlangen, Germany), Optima CT660 (GE Healthcare Japan Corporation, Hino, Japan), and uCT 780 (United Imaging, Shanghai, China). All examinations covered the entire thorax with patients in the supine position, both arms raised near the head, and at full inspiration; image acquisition extended from the thoracic inlet to below the costophrenic angles. The scanning parameters were as follows: tube voltage, 110–120 kVp; tube current, 50–140 mA (with automatic current modulation); section thickness, 5 mm; rotation time, 0.5 s; pitch, 1–1.1; collimation, 0.6 or 0.625 mm; reconstruction slice thickness and interval, 0.625 or 1 mm; and matrix, 512 × 512. Images were reconstructed with mediastinal (window width, 350–400 HU; level, 20–40 HU) and lung (window width, 1200–1600 HU; level, −500 to −700 HU) window settings.

### 2.3. Clinical Data and Image Analysis

Clinical data were obtained from the Electronic Medical Record System (Winning Health, Shanghai, China), which included patients’ age, gender, and smoking history; the pathological type of lesions; and tumor staging.

Thin-section CT (TSCT) images were analyzed on a PACS workstation using lung window (window width, 1200–1600 HU; level, −500 to −700 HU) and mediastinal window (window width, 350–400 HU; level, 20–40 HU) settings. All CT data were independently reviewed by two thoracic radiologists (X.-F.W. and X.-D.G., with 9 and 10 years of experience in radiology, respectively) who were blinded to clinical information and pathological diagnosis. Any interobserver discordance was resolved by reviewing the images together or by consulting a senior thoracic radiologist (Z.-G.C., with 15 years of experience in radiology) to reach consensus.

**Figure 2:**
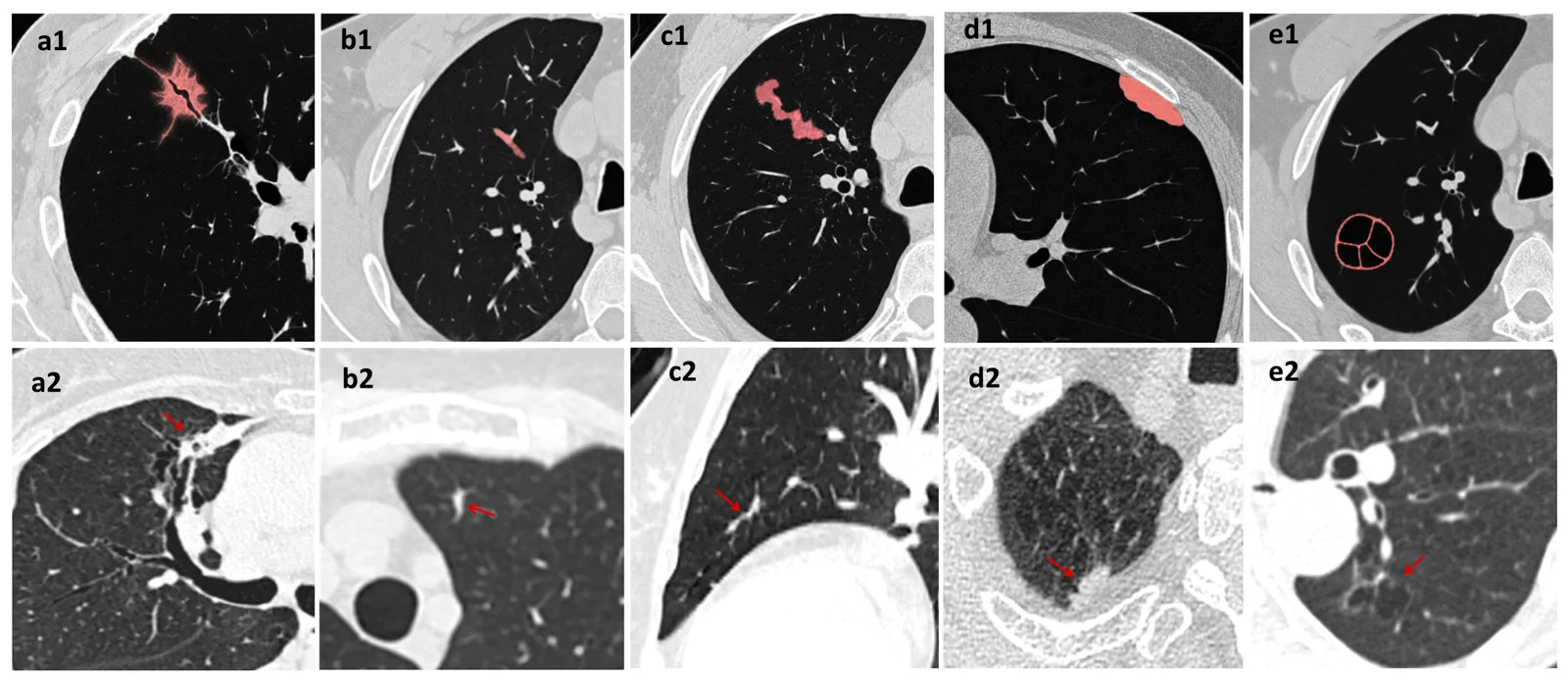
Schematic representations (**a1**–**e1**) and corresponding CT manifestations (**a2**–**e2**) of the non-nodular lesions. (**a1**,**a2**) Type I (patch-like), a localized patch-like opacity with an air bronchogram. (**b1**,**b2**) Type II (strip-like), an isolated short linear opacity with a well-defined boundary and smooth margin. (**c1**,**c2**) Type III (bead-like), a curvilinear opacity with serial focal bulges that produce a beaded contour. (**d1**,**d2**) Type IV (pleural plaque-like), a flat subpleural solid opacity abutting the pleura. (**e1**,**e2**) Type V (cystic-cavity), a multilocular cystic airspace with a thin wall and internal septa. Red arrows indicate the lesions in the CT images.

A nodule was defined as a circumscribed, typically round opacity no larger than 30 mm in average diameter [15], while other focal lesions that did not conform to nodular morphology were defined as non-nodular lesions. In this study, a non-nodular lesion was defined more narrowly as a focal pulmonary opacity that lacked a predominantly round or oval contour and matched one of the five patterns below; in addition, diffuse, segmental, or lobar opacities were not classified as non-nodular lesions. According to the morphological appearances, the non-nodular lesions were mainly classified into five subtypes (Figure 2): type I (patch-like), a focal solid or subsolid patchy opacity that may be accompanied by an air bronchogram; type II (strip-like), an isolated short strip or fusiform shadow with well-defined boundary, regular margin, and no pleural adhesion; type III (bead-like), an isolated continuous beaded or curvilinear opacity with irregular margin or focal bulges; type IV (pleural plaque-like), a focal flat subpleural solid opacity with a broad pleural base, with or without surrounding ground-glass opacity; and type V (cystic-cavity), a unilocular or multilocular cystic lesion with a uniformly thin and smooth wall or no wall.

The following features of nodules and non-nodular lesions were assessed on the initial CT scan: lobar location (upper, middle, or lower lobe), distribution relative to the pleura (subpleural or non-subpleural), density (solid or subsolid), boundary (well-defined or ill-defined), size (mean of the longest axial diameter and its perpendicular diameter), volume, spiculation, air bronchogram, vessel convergence, and pleural indentation.

The shortest distance between the lesion edge and the nearest pleural surface was measured and defined as the distance from the lesion edge to the pleura (D-ETP). Lesions with a D-ETP of 10 mm or less were classified as subpleural, whereas those with a D-ETP greater than 10 mm were classified as non-subpleural. Solid lesions were defined as showing homogeneous soft-tissue attenuation, whereas subsolid lesions contain ground-glass components, with or without solid components [15]. For cystic lesions, the density of the cyst wall was assessed. A well-defined boundary was defined as a clear tumor–lung interface; otherwise, it was considered ill-defined. Spiculation was defined as linear strands extending radially from the lesion surface into the surrounding lung parenchyma in a stellate manner [14]. Vessel convergence was defined as vascular structures converging toward the lesion, pleural indentation as a linear or strand-like connection between the lesion and adjacent pleura [14], and an air bronchogram as air-filled bronchi visualized within the lesion [14,16]. An artificial intelligence (AI) system (InferRead CT Lung, version 6.1.5.4; InferVision Medical Health, Beijing, China) was applied for semiautomatic volumetric measurement of lesions, with manual contour correction performed when necessary.

Volume doubling time (VDT) was calculated using the following equation:

(1) $$VDT=\frac{(t_{2}-t_{1})ln\;2}{ln(V_{2}/V_{1})}$$

where *V*_1_ and *V*_2_ represent lesion volumes at the initial (*t*_1_) and final (*t*_2_) CT examinations, respectively, and *t*_2_−*t*_1_ is the interval between examinations in days. VDT is expressed in days.

Concomitant pulmonary conditions associated with lung cancer risk [17,18,19] were also recorded, which included interstitial lung disease (ILD) (bilateral ground-glass or reticular abnormalities, architectural distortion, traction bronchiectasis or bronchiolectasis, and/or honeycombing in a distribution consistent with interstitial disease), emphysema (centrilobular, paraseptal, or panlobular subtype), and post-tuberculosis lung changes (fibrotic scars, calcifications, or pleural thickening consistent with prior tuberculosis).

### 2.4. Statistical Analysis

Statistical analyses were performed using R software (version 4.6.1; R Foundation for Statistical Computing, Vienna, Austria). Continuous variables were presented as mean ± standard deviation or median (interquartile range), and categorical variables as *n* (%). Depending on data distribution and the number of groups, continuous variables were compared using Student’s *t* test or one-way analysis of variance, or the Mann–Whitney U test or Kruskal–Wallis test. Categorical variables were compared using Pearson’s chi-square test or Fisher’s exact test; Monte Carlo or conditional permutation *p* values were used for sparse five-subtype tables. Significant overall subtype differences were followed by subtype-versus-rest Fisher’s exact tests with Holm correction. Interobserver agreement was assessed using the intraclass correlation coefficient (ICC) for continuous variables and Cohen’s kappa or Fleiss’ kappa for categorical variables. ICC values were classified as poor (<0.50), moderate (0.50–0.74), good (0.75–0.89), or excellent (≥0.90). Kappa values were classified as slight (≤0.20), fair (0.21–0.40), moderate (0.41–0.60), substantial (0.61–0.80), or almost perfect (≥0.81). Variables representing patient characteristics, lesion distribution, and concomitant pulmonary diseases were prespecified a priori for Firth penalized-likelihood logistic regression, with conventional logistic regression used as a sensitivity analysis. Multicollinearity was assessed using generalized variance inflation factors (GVIFs) and dimension-adjusted GVIF^1/(2 × Df)^ values, where Df denotes the degrees of freedom. Adjusted odds ratios with 95% confidence intervals were reported. The area under the receiver operating characteristic curve (AUC), with a DeLong 95% confidence interval (CI), was calculated as a descriptive measure of separation between the nodular and non-nodular groups. All tests were two-sided, and *p* < 0.05 indicated statistical significance.

## 3. Results

### 3.1. Interobserver Agreement for CT Features

Interobserver agreement was good to excellent for lesion size and volume (ICC range, 0.891–0.925). For categorical features, agreement was almost perfect for non-nodular subtype assignment [type I (patch-like), type II (strip-like), type IV (pleural plaque-like), and type V (cystic-cavity)], D-ETP, density, air bronchogram, and pleural indentation (κ = 0.813–0.891), and substantial for type III (bead-like) assignment, boundary, spiculation, and vessel convergence (κ = 0.707–0.775) (Table S2).

### 3.2. Patients’ Characteristics and CT Features of the Five Non-Nodular Subtypes

Of the 365 SLCs, 258 (70.68%) initially presented as nodules and 107 (29.32%) as non-nodular lesions. On initial CT images, non-nodular lesions were more frequently misinterpreted as various benign abnormalities than nodules (71.00% vs. 32.20%) but were less frequently suspected to be lung cancers (3.70% vs. 34.50%) (all *p* < 0.001) (Table S1).

**Figure 3:**
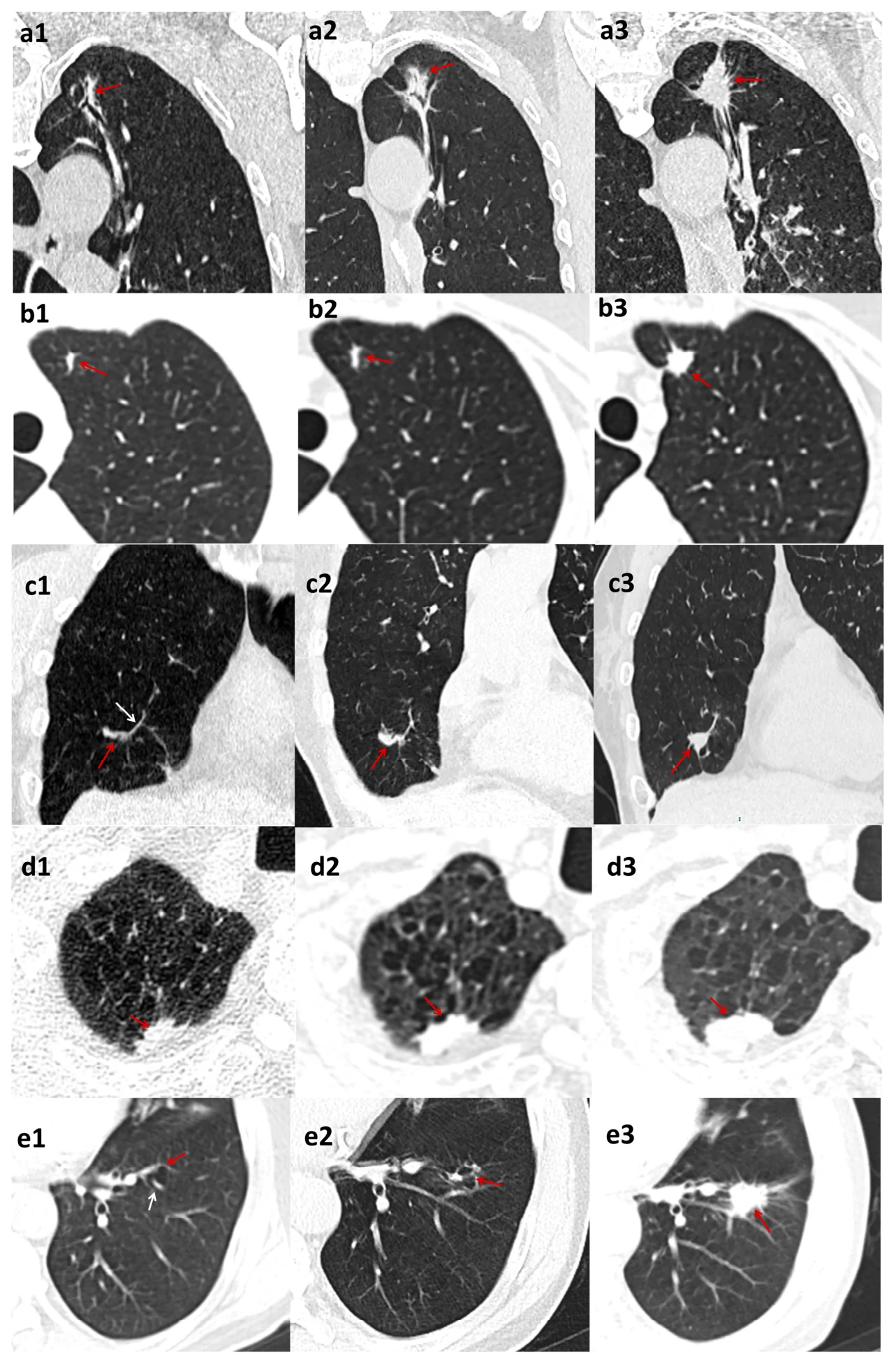
Serial CT evolution of five non-nodular patterns of solid lung cancer. (**a1**–**a3**) Type I (patch-like), left upper lobe: enlargement with an air bronchogram and spiculation at 18 months and progression to a solid mass at 22 months. (**b1**–**b3**) Type II (strip-like), left upper lobe: enlargement at 8 months and a solid nodule with spiculation and pleural indentation at 15 months. (**c1**–**c3**) Type III (bead-like), right lower lobe: slight enlargement at 18 months and a solid nodule at 30 months. (**d1**–**d3**) Type IV (pleural plaque-like), right upper lobe: enlargement at 9 months and further growth with lobulation at 15 months. (**e1**–**e3**) Type V (cystic-cavity), left lower lobe: focal wall thickening at 18 months and a solid nodule at 24 months. Red arrows indicate lesions; white arrows indicate vessel convergence (**c1**) and a traversing pulmonary venous branch (**e1**). Histopathology confirmed squamous cell carcinoma in (**b1**–**b3**) and invasive adenocarcinoma in the other four lesions. CT, computed tomography.

The patients’ clinical characteristics and pathological and CT findings of lesions in the non-nodular group are shown in Table 1. Among the 107 non-nodular lesions, type I (patch-like) was the most common (56, 52.34%), followed by type III (bead-like) (20, 18.69%), type II (strip-like) (16, 14.95%), type IV (pleural plaque-like) (9, 8.41%), and type V (cystic-cavity) (6, 5.61%). All subtypes were predominantly located in the upper lobes (50.00%–83.33%) and distributed within the subpleural region (56.25%–100.00%). Compared with the other four subtypes combined, type I (patch-like) lesions more often showed subsolid density, ill-defined boundaries, spiculation, air bronchograms, vessel convergence, and pleural indentation (all Holm-adjusted *p* < 0.001); type II (strip-like) lesions were more often solid and well defined. Both type II and type III (bead-like) lesions showed less frequent spiculation and pleural indentation than the other four subtypes combined (all Holm-adjusted *p* ≤ 0.032). Type IV (pleural plaque-like) lesions were uniformly subpleural, solid, and well defined. Type V (cystic-cavity) lesions mainly manifested as multilocular cystic airspaces with thin walls and internal septa (Figure 3).

The median VDTs for types I (patch-like), II (strip-like), III (bead-like), IV (pleural plaque-like), and V (cystic-cavity) were 346.84, 317.22, 428.89, 217.84, and 424.98 days, respectively (overall Kruskal–Wallis *p* = 0.131).

### 3.3. Clinical and Pathological Characteristics of the Nodular and Non-Nodular Groups

The patients’ clinical characteristics are summarized in Table 2. Compared with the nodular group, patients in the non-nodular group were older (65.00 ± 8.34 vs. 62.34 ± 9.42 years; *p* = 0.011), with a higher proportion of males (75.70% vs. 58.53%; *p* = 0.002) and smokers (59.81% vs. 47.29%; *p* = 0.029). The distribution of pathological types differed between the groups (*p* = 0.005). Adenocarcinoma predominated in both groups, whereas the proportion of squamous cell carcinoma was higher in the non-nodular group (24.30% vs. 13.57%).

In the non-nodular group, pathological diagnosis was established by bronchoscopic sampling in 7 patients (6.54%), percutaneous needle biopsy in 6 (5.61%), and histopathological examination of surgical resection specimens in 94 (87.85%). The corresponding numbers in the nodular group were 39 (15.12%), 22 (8.53%), and 197 (76.36%), respectively.

### 3.4. CT Features and Longitudinal Changes in the Nodular and Non-Nodular Groups

The CT features of lesions in the two groups are summarized in Table 3. Both nodules and non-nodular lesions were most frequently located in the upper lobes (55.04% vs. 58.88%, *p* = 0.611). Compared with nodules, non-nodular lesions were more frequently located in the subpleural region (81.31% vs. 65.12%; *p* = 0.002), and more often exhibited subsolid density (35.51% vs. 24.42%; *p* = 0.031), ill-defined boundary (39.25% vs. 5.81%; *p* < 0.001), and pleural indentation (35.51% vs. 21.71%; *p* = 0.006). Additionally, concomitant pulmonary emphysema (67.29% vs. 28.68%), ILD (56.07% vs. 2.33%), and post-tuberculosis lung changes (14.02% vs. 2.33%) were more common in the non-nodular group (all *p* < 0.001).

**Figure 4:**
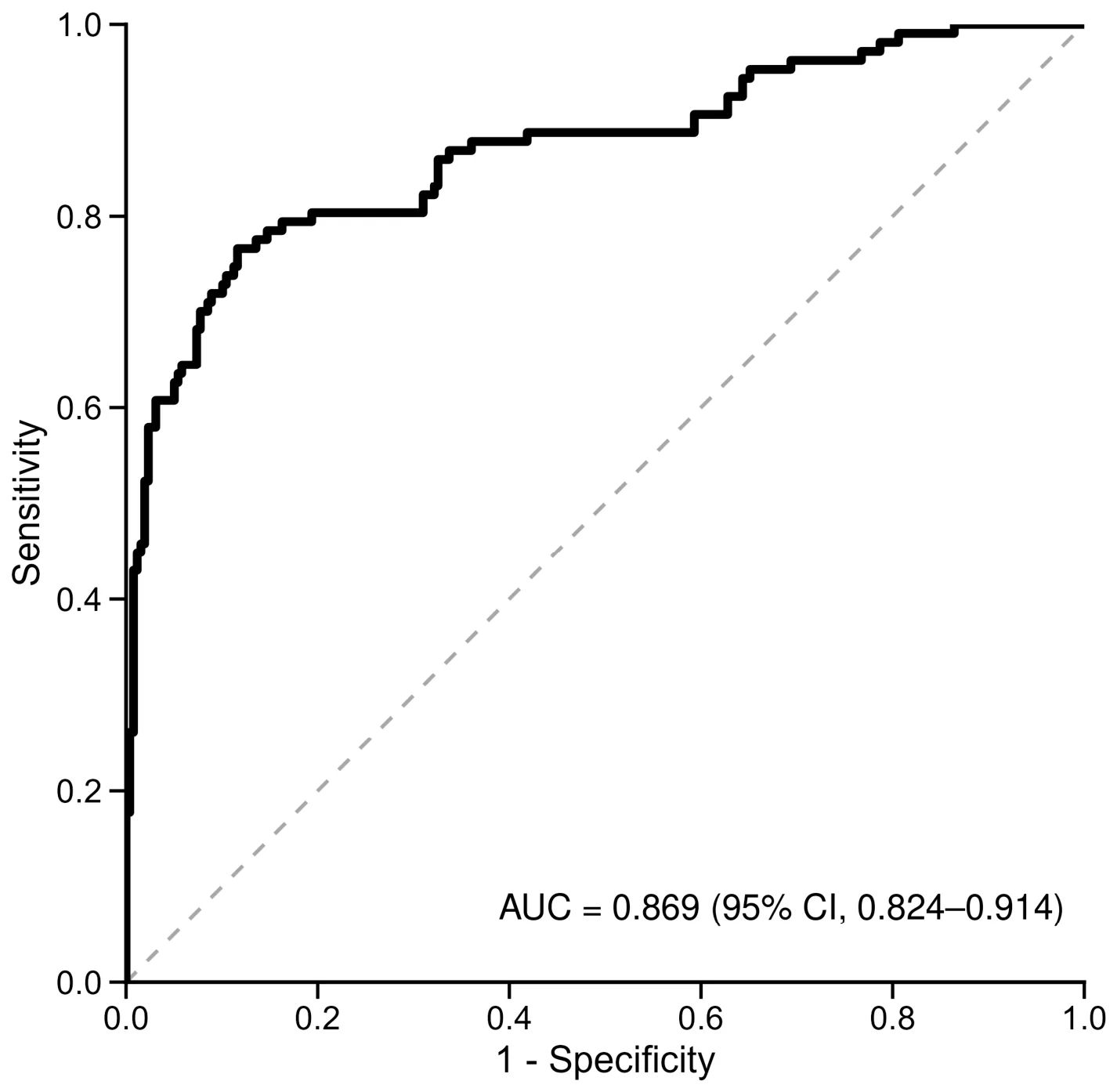
Receiver operating characteristic curve based on factors independently associated with a non-nodular pattern. The curve descriptively summarizes separation between the nodular and non-nodular groups. The solid black line represents the model ROC curve, and the dashed gray diagonal indicates chance-level discrimination (AUC = 0.5). AUC, area under the curve; CT, computed tomography.

Table 4 presents the factors independently associated with a non-nodular pattern in the Firth multivariable analysis. The GVIFs ranged from 1.080 to 2.797, with dimension-adjusted GVIFs ranging from 1.024 to 1.672, indicating no substantial multicollinearity among the included covariates (Table S3). Smoking history (adjusted odds ratio [aOR], 2.93; 95% confidence interval [CI], 1.05–8.28; *p* = 0.040), subpleural distribution (aOR, 3.37; 95% CI, 1.61–7.72; *p* < 0.001), ILD (aOR, 66.45; 95% CI, 23.57–220.04; *p* < 0.001), and post-tuberculosis lung changes (aOR, 8.60; 95% CI, 2.37–32.64; *p* = 0.001) were independently associated with a non-nodular pattern. The ROC curve derived from the full multivariable model yielded an AUC of 0.869 (95% CI, 0.824–0.914; *p* < 0.001) (Figure 4).

The lesions in the two groups exhibited comparable volumes on the initial CT and similar volume doubling times; however, non-nodular lesions had a longer follow-up interval (734.00 [416.00–1068.00] vs. 377.50 [213.00–742.30] days; *p* < 0.001) and were larger on the last CT in both diameter (19.00 [13.50–29.00] vs. 15.50 [12.00–24.00] mm; *p* = 0.002) and volume (3049.12 [1170.20–7306.10] vs. 1711.16 [697.80–6351.90] mm^3^; *p* = 0.005).

## 4. Discussion

In this selected cohort, 107 of 365 SLCs (29.32%) initially manifested as non-nodular lesions on CT scans. Among these non-nodular lesions, patch-like lesions were the most prevalent, followed by bead-like, strip-like, pleural plaque-like, and cystic-cavity lesions. Compared with nodules, non-nodular lesions showed a similar upper-lobe predominance but were more frequently located in subpleural regions. In smokers and patients with ILD or post-tuberculosis lung changes, subpleural non-nodular lesions identified in the upper lobes warrant close surveillance. The present findings expand current understanding of early CT manifestations of SLCs and facilitate timely lesion recognition and targeted monitoring, thereby enabling prompt therapeutic intervention.

Review of the initial CT data showed that 71.00% of non-nodular lesions were interpreted as inflammation or other benign lesions, and only 3.70% were suspected to be malignant, compared with 34.50% of nodular lesions. Owing to their atypical morphology, non-nodular lesions may attract insufficient attention and are more likely to be recognized belatedly. In fact, compared with nodules, these non-nodular lesions had longer follow-up intervals and were larger at the time of definitive diagnosis. Accordingly, it is necessary to reveal the characteristics of non-nodular lesions for early identification. Although nodular and non-nodular lesions differed morphologically, their comparable VDTs indicated similar growth rates. Therefore, the five non-nodular subtypes represent a heterogeneous morphological spectrum rather than a separate biological subtype. Type II (strip-like) and type III (bead-like) lesions merit particular attention because they were frequently well defined and lacked spiculation and pleural indentation, which may make them resemble benign linear abnormalities. Type V (cystic-cavity) overlaps with previously described lung cancers associated with cystic airspaces [20,21] and included only six cases. Larger studies are needed to establish the diagnostic and clinical significance of each subtype.

On the initial CT, non-nodular lesions were frequently misdiagnosed because they often mimicked common benign abnormalities. Patch-like lesions were frequently interpreted as focal inflammation owing to their subsolid density, ill-defined boundary, and consolidation-like appearance. Strip-like and bead-like lesions may be mistaken for fibrosis or post-inflammatory scars, as they were relatively localized and frequently lacked other characteristic CT features of SLCs. When extending along a small bronchus, a strip-like lesion could also resemble mucus plugging or mucoid impaction [22,23]. Pleural plaque-like lesions exhibited overlapping features with focal pleural thickening and subpleural fibrosis, findings also commonly observed in the upper lobes. Additionally, in patients with emphysema, cystic-cavity lesions might be misidentified as simple bullae, particularly when their walls were thin or indistinct. Thus, these subtypes share imaging similarities with their benign counterparts, necessitating careful differential evaluation.

Nevertheless, some CT features of non-nodular lesions may facilitate differentiation. In patch-like lesions, bronchial distortion or traction bronchiectasis within the opacity, together with persistence on follow-up, should raise suspicion of malignancy, as the bronchi typically remain normal in inflammatory lesions. Strip-like and bead-like lesions are typically isolated and lack pleural attachment, distinguishing them from common fibrous scars. Pleural plaque-like lesions tend to be unilateral and localized, and in some cases an acute angle can be observed between the lesion and the pleura, whereas benign apical caps or subpleural fibrosis are generally bilateral [14] and often form an obtuse angle with the pleura. Cystic-cavity lesions are more often multilocular and may have thicker walls than simple bullae, although they may lack the irregular wall thickening or mural nodularity typically seen in lung cancers associated with cystic airspaces. New or progressive wall thickening, mural nodularity, or an enlarging solid component on follow-up should therefore prompt heightened suspicion [20,21,24]. Since these differences may be subtle on a single examination, close surveillance and careful comparison with prior CT scans are essential to identify interval progression and allow early recognition of these atypical lung cancers.

The exploratory subtype VDT findings do not establish subtype-specific follow-up intervals. For selected indeterminate lesions suitable for surveillance, particularly type I patch-like abnormalities that may reflect inflammation, CT at approximately 3 months may help assess persistence. If a lesion persists but remains stable and of low suspicion after reassessment, a further CT approximately 6 months after that examination may be considered. Lung-RADS v2022 provides screening-based clinical context for these suggestions [25], which were not validated by this cohort. Growing or otherwise suspicious lesions warrant prompt diagnostic evaluation.

Previous studies have shown that lung cancer is more common in the upper lobes [26]. Consistent with these findings, both nodular and non-nodular cancers in this cohort were predominantly located in the upper lobes. However, non-nodular lesions were more often subpleural, suggesting that persistent non-nodular abnormalities in the subpleural regions of the upper lobes require special attention. Smoking history was also more common in the non-nodular group. Given the well-known association between smoking and squamous cell carcinoma (SCC) [27], this finding may partly explain the higher proportion of SCC in this group. Among non-nodular lesions, SCC mainly occurred in bead-like, strip-like, and patch-like subtypes. Pathologically, peripheral SCC may grow by filling alveolar spaces [28] or, in rare cases, extend along the lumen of distal bronchi [29]. These growth patterns may lead to a noncompact appearance and partly explain the strip-like or bead-like morphology of some peripheral SCCs.

Beyond lesion distribution and smoking history, background lung disease was also closely related to initial lesion morphology. In this study, ILD and post-tuberculosis lung changes were independently associated with a non-nodular pattern. Fibrosis, traction bronchiectasis, scarring, and pleural thickening alter lung architecture and may partly affect the CT appearance of early tumors, resulting in linear, plaque-like, or other non-nodular appearances [30,31,32,33]. This finding highlights the importance of comparing new or asymmetric focal abnormalities with prior CT scans, especially when these lesions develop within a complex pulmonary background. Both participating centers are located in Chongqing, southwestern China, where a substantial tuberculosis burden has been documented [34] and pulmonary cryptococcosis has been reported in local clinical practice [35]. This background underscores the importance of considering malignancy in persistent or progressive non-nodular abnormalities that resemble infection or post-inflammatory scarring.

This study has several limitations. First, the retrospective design and enrollment of only pathologically confirmed SLCs with sufficient longitudinal CT data may introduce selection bias. The reduction from the source population mainly reflects our focus on peripheral lung cancers with a pure solid appearance on the latest CT and sufficient longitudinal imaging. Patients with lesions that remained subsolid or with a shorter CT interval were outside these eligibility criteria. These restrictions may underrepresent rapidly growing or promptly treated cancers and limit generalizability to the broader lung cancer population. Second, the small sample sizes for certain non-nodular subtypes limit the precision of comparisons among subtypes. Third, the classification system for non-nodular lesions proposed in this study has rarely been reported, so external validation is needed in future investigations. Fourth, no benign comparison group was included. We did not evaluate the serial CT appearances of benign lesions, so their morphological overlap with early SLC remains unknown. These findings do not establish the specificity of the non-nodular patterns reported here. Finally, this analysis mainly focused on morphological characteristics, while contrast-enhanced CT findings and other quantitative imaging biomarkers were not assessed.

## 5. Conclusions

Besides nodules, SLCs in the early growth phase may present as diverse non-nodular abnormalities, including patch-like, strip-like, bead-like, pleural plaque-like, and cystic-cavity patterns. In smokers or patients with ILD or post-tuberculosis lung changes, subpleural non-nodular lesions in the upper lobes warrant greater attention and close follow-up for interval growth. Broadening the recognized spectrum of early SLCs may help reduce diagnostic delay and extend early detection beyond a nodule-centered approach.

## Figures and Tables

**Table 1:** Clinical, pathological, and CT characteristics of the five non-nodular subtypes.

Variables	Subtypes of Non-Nodular Lesions					*p* Value
	Type I (Patch-Like) (*n* = 56)	Type II (Strip-Like) (*n* = 16)	Type III (Bead-Like) (*n* = 20)	Type IV (Pleural Plaque-Like) (*n* = 9)	Type V (Cystic-Cavity) (*n* = 6)	
Demographics						
Age (years)	64.66 ± 8.51	66.12 ± 9.27	66.65 ± 7.65	61.67 ± 8.02	64.67 ± 7.69	0.632 ^a^
Gender (male)	43 (76.79)	13 (81.25)	15 (75.00)	6 (66.67)	4 (66.67)	0.883 ^c^
Smoking history (smoker)	36 (64.29)	9 (56.25)	10 (50.00)	5 (55.56)	4 (66.67)	0.814 ^c^
Pathological type						0.033 ^c^
Adenocarcinoma	39 (69.64)	8 (50.00)	12 (60.00)	6 (66.67)	5 (83.33)	
Squamous cell carcinoma	14 (25.00)	5 (31.25)	7 (35.00)	0 (0.00)	0 (0.00)	
Neuroendocrine carcinomas	0 (0.00)	1 (6.25)	1 (5.00)	0 (0.00)	0 (0.00)	
Others	3 (5.36)	2 (12.50)	0 (0.00)	3 (33.33)	1 (16.67)	
Lobar location						0.610 ^c^
Upper lobe	33 (58.93)	10 (62.50)	10 (50.00)	5 (55.56)	5 (83.33)	
Middle lobe	6 (10.71)	1 (6.25)	1 (5.00)	0 (0.00)	0 (0.00)	
Lower lobe	17 (30.36)	5 (31.25)	9 (45.00)	4 (44.44)	1 (16.67)	
CT pattern						<0.001 ^c^
Solid	26 (46.43)	16 (100.00)	12 (60.00)	9 (100.00)	6 (100.00)	
Subsolid	30 (53.57) ^†^	0 (0.00) ^‡^	8 (40.00)	0 (0.00)	0 (0.00)	
D-ETP						0.063 ^c^
>10 mm	8 (14.29)	7 (43.75)	4 (20.00)	0 (0.00)	1 (16.67)	
≤10 mm	48 (85.71)	9 (56.25)	16 (80.00)	9 (100.00)	5 (83.33)	
Boundary						<0.001 ^c^
Ill-defined	38 (67.86) ^†^	0 (0.00) ^‡^	4 (20.00)	0 (0.00) ^‡^	0 (0.00)	
Well-defined	18 (32.14)	16 (100.00)	16 (80.00)	9 (100.00)	6 (100.00)	
Spiculation	23 (41.07) ^†^	0 (0.00) ^‡^	0 (0.00) ^‡^	3 (33.33)	0 (0.00)	<0.001 ^c^
Air bronchogram	31 (55.36) ^†^	0 (0.00) ^‡^	5 (25.00)	0 (0.00)	0 (0.00)	<0.001 ^c^
Vessel convergence	26 (46.43) ^†^	0 (0.00) ^‡^	4 (20.00)	2 (22.22)	0 (0.00)	<0.001 ^c^
Pleural indentation	38 (67.86) ^†^	0 (0.00) ^‡^	0 (0.00) ^‡^	0 (0.00)	0 (0.00)	<0.001 ^c^

Note: Data are presented as mean ± standard deviation for age and as *n* (%) for categorical variables. Overall *p* values were obtained using one-way analysis of variance for age and Monte Carlo or conditional permutation tests for categorical variables. Superscripts † and ‡ indicate frequencies significantly higher and lower, respectively, than those in the other four subtypes combined after two-sided Fisher’s exact tests with Holm correction. Types I–V represent patch-like, strip-like, bead-like, pleural plaque-like, and cystic-cavity lesions, respectively. ^a^ calculated by Student’s *t* test or one-way analysis of variance, as appropriate. ^c^ calculated by Fisher’s exact test or a Monte Carlo exact test, as appropriate.

**Table 2:** Clinical characteristics of patients in the nodular and non-nodular groups.

Variables	Nodular Group (*n* = 258)	Non-Nodular Group (*n* = 107)	*p* Value
Age at initial CT (years)	62.34 ± 9.42	65.00 ± 8.34	0.011 ^a^
Gender			0.002 ^b^
Male	151 (58.53)	81 (75.70)	
Female	107 (41.47)	26 (24.30)	
Smoking history			0.029 ^b^
Yes	122 (47.29)	64 (59.81)	
No	136 (52.71)	43 (40.19)	
Pathological type			0.005 ^b^
Adenocarcinoma	192 (74.42)	70 (65.42)	
Squamous cell carcinoma	35 (13.57)	26 (24.30)	
Neuroendocrine carcinomas	20 (7.75)	2 (1.87)	
Others	11 (4.26)	9 (8.41)	
Tumor stage at final diagnosis			0.160 ^b^
Stage I	192 (74.42)	70 (65.42)	
Stage II	21 (8.14)	10 (9.35)	
Stage III	27 (10.47)	12 (11.21)	
Stage IV	18 (6.98)	15 (14.02)	

Note: Values are expressed as number (%) or mean ± SD. ^a^ calculated by Student’s *t* test or one-way analysis of variance, as appropriate. ^b^ calculated by Pearson’s chi-square test.

**Table 3:** CT features and longitudinal changes in the nodular and non-nodular groups.

Variable	Nodular Group (*n* = 258)	Non-Nodular Group (*n* = 107)	*p* Value
Distribution			0.611 ^b^
Upper lobe	142 (55.04)	63 (58.88)	
Middle lobe	18 (6.98)	8 (7.48)	
Lower lobe	98 (37.98)	36 (33.64)	
Location			0.002 ^b^
D-ETP > 10 mm	90 (34.88)	20 (18.69)	
D-ETP ≤ 10 mm	168 (65.12)	87 (81.31)	
Density pattern			0.031 ^b^
Solid	195 (75.58)	69 (64.49)	
Subsolid	63 (24.42)	38 (35.51)	
Boundary			<0.001 ^b^
Ill-defined	15 (5.81)	42 (39.25)	
Well-defined	243 (94.19)	65 (60.75)	
Pleural indentation	56 (21.71)	38 (35.51)	0.006 ^b^
Concomitant pulmonary disease			
Emphysema	74 (28.68)	72 (67.29)	<0.001 ^b^
Interstitial lung disease	6 (2.33)	60 (56.07)	<0.001 ^b^
Post-tuberculosis lung changes	6 (2.33)	15 (14.02)	<0.001 ^b^
Volume on initial CT (mm^3^) ^#^	553.44 (208.10, 1469.00)	563.60 (228.50, 1165.80)	0.642 ^d^
Diameter on last CT (mm) ^#^	15.50 (12.00, 24.00)	19.00 (13.50, 29.00)	0.002 ^d^
Volume on last CT (mm^3^) ^#^	1711.16 (697.80, 6351.90)	3049.12 (1170.20, 7306.10)	0.005 ^d^
Follow-up interval (days) ^#^	377.50 (213.00, 742.30)	734.00 (416.00, 1068.00)	<0.001 ^d^
VDT (days) ^#^	311.73 (168.40, 706.50)	332.12 (182.20, 479.30)	0.655 ^d^

Note: Values are *n* (%) or median (interquartile range). CT, computed tomography; D-ETP, distance from the lesion edge to the pleura; VDT, volume doubling time. ^#^ data are presented as median (interquartile range [IQR]). ^b^ calculated by Pearson’s chi-square test. ^d^ calculated by the Mann–Whitney U test or Kruskal–Wallis H test, as appropriate.

**Table 4:** Multivariable Firth logistic regression for factors associated with a non-nodular pattern.

Variables	Adjusted Odds Ratio (95% CI)	*p* Value
Age, years	1.00 (0.97–1.04)	0.873 ^e^
Sex		0.783 ^e^
Female	1	
Male	1.13 (0.45–2.71)	
Smoking history		0.040 ^e^
Smoker	2.93 (1.05–8.28)	
Non-smoker	1	
Lobar distribution		0.631 ^e^
Upper lobe	1	
Middle lobe	1.68 (0.52–4.93)	
Lower lobe	0.97 (0.50–1.85)	
Subpleural location		<0.001 ^e^
No	1	
Yes	3.37 (1.61–7.72)	
Pulmonary emphysema		0.497 ^e^
No	1	
Yes	1.38 (0.54–3.39)	
Interstitial lung disease		<0.001 ^e^
No	1	
Yes	66.45 (23.57–220.04)	
Post-tuberculosis lung changes		0.001 ^e^
No	1	
Yes	8.60 (2.37–32.64)	

Firth logistic regression used a non-nodular pattern as the outcome; reference categories have an odds ratio of 1. The lobar-distribution *p* value is the overall penalized likelihood-ratio test. CI, confidence interval. ^e^ calculated using Firth penalized-likelihood logistic regression.

## Data Availability

The datasets generated during the current study are not publicly available due to our institutional regulations but are available from the corresponding author on reasonable request.

## Supplementary Materials

The following supporting information can be downloaded at: https://www.mdpi.com/article/10.3390/diagnostics16203332/s1. Table S1: Initial CT diagnosis of lesions in the nodular and non-nodular groups; Table S2: Interobserver agreement for classifications of non-nodular lesions and CT features of all lesions; Table S3: Generalized variance inflation factors for covariates in the multivariable Firth logistic regression model.
