# Supplementary material for: Non-Nodular CT Patterns of Solid Lung Cancer in the Early Growth Phase: A Two-Center Retrospective Study
Source: Diagnostics (Basel). 2026 Oct 14;16(20):3332. doi: 10.3390/diagnostics16203332 (PMC13703298; doi:10.3390/diagnostics16203332)
Supplement: Supplementary file 1 [file diagnostics-16-03332-s001.zip › diagnostics-4574743-supplementary.pdf]

***Table S1.* Initial CT diagnosis of lesions in the nodular and non-nodular groups**

Initial diagnosis	Nodular group (n = 258)	Non-nodular group (n = 107)	P value
Neoplastic lesions	89 (34.50)	4 (3.74)	< 0.001[b]
Indeterminate lesions	86 (33.3)	27 (25.2)	
Inflammatory or other benign lesions	83 (32.2)	76 (71.0)	

Notes: Data are expressed as number (percentage).
[b] Calculated by Pearson's chi-square test.

***Table S2*. Interobserver agreement for classifications of non-nodular lesions and CT features of all lesions**

CT features	Metric	95% CI	P-value
Type I of non-nodular lesions	0.885	0.764 – 0.972	< 0.001
Type II of non-nodular lesions	0.829	0.745 – 0.917	< 0.001
Type III of non-nodular lesions	0.723	0.526 – 0.880	< 0.001
Type IV of non-nodular lesions	0.835	0.764 – 0.965	< 0.001
Type V of non-nodular lesions	0.850	0.797 – 0.955	< 0.001
D-ETP	0.891	0.756 – 1.000	< 0.001
Density	0.884	0.747 – 1.000	< 0.001
Boundary	0.707	0.615 – 0.799	< 0.001
Spiculation	0.767	0.640 – 0.871	< 0.001
Air bronchogram sign	0.813	0.748 – 0.878	< 0.001

Vessel convergence	0.775	0.572 – 0.949	< 0.001
Pleural indentation	0.823	0.696 – 1.000	< 0.001
Diameter	0.925	0.898 – 0.941	< 0.001
Volume on initial CT	0.891	0.823 – 0.959	< 0.001
Volume on last CT	0.902	0.871 – 0.948	< 0.001

Metric represents intraclass correlation coefficient for continuous variables and kappa coefficient for categorical variables. CI, confidence interval; D-ETP, distance from lesion edge to pleura

Table S3. **Generalized variance inflation factors for covariates in the multivariable Firth logistic regression model**

Variables	GVIF	Df	$GVIF^{1/(2 \times Df)}$
Age, years	1.080	1	1.039
Sex	2.245	1	1.498
Smoking history	2.797	1	1.672
Lobar distribution	1.101	2	1.024
Subpleural location	1.089	1	1.043
Pulmonary emphysema	2.115	1	1.454
Interstitial lung disease	1.565	1	1.251
Post-tuberculosis lung changes	1.202	1	1.096

GVIF, generalized variance inflation factor; Df, degrees of freedom. The dimension-adjusted GVIF is calculated as $GVIF^{1/(2 \times Df)}$.

Values were calculated from the coefficient correlation matrix obtained from the inverse expected Fisher information evaluated at the Firth estimates, excluding the intercept (n = 365). Lobar distribution was modeled as a three-level factor (upper, middle, and lower lobes; Df = 2); all other terms had Df = 1.
